# The Public Perception of Zoophilic Acts in Hungary

**DOI:** 10.3390/ani15040465

**Published:** 2025-02-07

**Authors:** Szilvia Vetter, Beáta Szilassi, László Ózsvári

**Affiliations:** 1 Centre for Animal Welfare, University of Veterinary Medicine Budapest, 1078 Budapest, Hungary; szilassi.bea@gmail.com; 2 Department of Veterinary Forensics and Economics, University of Veterinary Medicine Budapest, 1078 Budapest, Hungary; ozsvari.laszlo@univet.hu

**Keywords:** animal welfare, zoophilia, zoophile acts, animal cruelty, penal sanctioning

## Abstract

This study aimed to assess public perceptions and knowledge of, and attitudes towards, zoophilia in Hungary. Conducted between October and December 2021 with 1753 respondents, the survey revealed significant interest and concern regarding zoophilia. The majority (98.3%) of respondents deemed zoophilia to be unacceptable from both health and animal welfare perspectives. Of those surveyed, 98.9% believed that animals possess dignity, and 84.7% felt that zoophilia negatively impacts this dignity. However, awareness of Hungarian legislation on zoophilia was limited, with 38.9% of respondents unaware that such acts are legally prohibited. The survey also highlighted a strong consensus (98.2%) favoring strict penalties for zoophilia. Additionally, the survey uncovered that 14% of respondents had encountered or heard of zoophilic incidents, involving various animals, predominantly dogs. Gender and settlement type significantly influence attitudes toward zoophilic acts, with women and people in capital and major cities being more aware of the legal prohibition and more supportive of strict sanctions. The results underscore a crucial need for enhanced public education on legal standards and ethical considerations regarding zoophilia and suggest a significant gap in understanding and managing the issue, necessitating further research and legislative reforms.

## Simple Summary

The study examined public attitudes towards zoophilia in Hungary, surveying 1753 people. It revealed strong disapproval of zoophilia, with most respondents condemning it due to health and animal welfare concerns. Many recognized animals’ dignity and believed that zoophilia harms it. However, awareness of Hungary’s legal stance was limited, with a significant number unaware of its prohibition. Most respondents supported strict penalties for zoophilic acts. Among those who reported encountering such incidents, the majority involved dogs. Gender and settlement type affect attitudes toward zoophilic acts, with women and city residents showing higher awareness and support for strict sanctions. The findings highlight the need for better public education and legal reforms.

## 1. Introduction

Zoophilia, defined as a paraphilic interest in animals involving sexual attraction or activity [1], has been the subject of psychological, ethical and legal discourse. Although zoophilia largely remains a taboo subject in contemporary times, it has been documented throughout human history and reflects a complex interplay of cultural, legal, and ethical dimensions [2,3]. Evidence of such phenomena dates back to prehistoric times; according to some authors, human–animal sexual interactions occurred between 40,000 and 25,000 years ago [4]. Depictions and references appeared in ancient cultures. In Ancient Egypt, artifacts and hieroglyphics indicate practices and beliefs related to sexual relations with animals [5]. Greek and Roman mythology frequently featured gods transforming into animals to seduce humans, a motif symbolizing the intertwining of human and animal realms. However, these acts were often met with severe condemnation. Ancient legal codes, such as the Code of Hammurabi, prescribed harsh penalties for bestiality, illustrating a shift towards stricter moral and legal standards [3]. Throughout the Middle Ages, while zoophilia was sometimes thought to possess medicinal qualities, it was also associated with witchcraft and heresy, leading to severe punishments for those involved [4,6].

The Renaissance period saw a resurgence of interest and, in some cases, legal tolerance towards zoophilia, though it remained controversial [7]. Notable incidents, including the use of turkeys in Parisian brothels, underscore the persistent moral ambiguity surrounding these practices [8]. The 19th and early 20th centuries brought a shift towards decriminalization in some regions, influenced by evolving attitudes towards sexual behaviors and legal reforms. Despite this, the widespread acknowledgment of zoophilia’s prevalence, particularly in rural communities, as noted by Kinsey’s research [9], emphasizes its persistence across cultures and time periods. For much of Europe’s modern history, zoophilia was decriminalized, partly due to the separation of ethics from law, and by the mid-20th century, 80% of European countries did not penalize zoophilic acts [10]. However, in the past 10–15 years, this trend has reversed. To protect both humans and animals, zoophilia has been re-criminalized in most European nations, usually carrying penalties of several years’ imprisonment [11]. In the last two decades, a number of works have emerged that seek to assess the complexities of bestiality and zoophilia from a multidisciplinary perspective. For example, in the edited study volume that they compiled, Beetz and Podberscek (2005) provided a platform that integrated insights from various disciplines, including psychology, law, veterinary medicine, and sociology [12]. Nowadays, the internet plays a significant role in connecting individuals with zoophilic interests, fostering communities that share experiences and information [13,14].

Zoophilia, often classified as a paraphilia, involves a persistent and intense sexual attraction to animals. According to the American Psychiatric Association, this condition is characterized by recurrent sexual arousal primarily directed toward animals, and it may qualify as a disorder when it causes significant distress or impairment [15]. Despite the growing number of new studies and research works emerging on this topic, bestiality can still be regarded as an under-researched area within human sexuality. Fundamental questions about its lifetime prevalence, motivations, and relationships with psychological health remain largely unanswered, underscoring the need for further investigation to deepen our understanding of this complex issue [16]. Efforts to categorize zoophilia have led to various classification systems. Aggrawal’s framework, for example, distinguishes between different types of zoophilic behaviors based on their nature and intensity. In the realm of zoophilia, behaviors can be broadly categorized into three main types. Non-contact behaviors involve activities such as fantasies or voyeuristic interactions with animals, where no physical contact is present. Contact behaviors encompass physical interactions with animals, ranging from petting to more invasive actions. Importantly, from an animal welfare and protection perspective, only contact behaviors involving actual physical interaction with living animals have direct relevance. Additionally, zoosadism is a specific form where individuals derive sexual pleasure from inflicting pain or suffering on animals, representing an extreme and troubling aspect of zoophilia with significant ethical and legal implications [1]. Consequently, it can be deduced that not all categories of zoophilia carry potential relevance for animal protection. A distinction must be made between zoophilia and zoophilic acts. Essentially, while zoophilia denotes psychological or emotional engagement, zoophilic acts involve concrete and often ethically or legally troubling physical interactions with animals. The legal relevance pertains specifically to zoophilic acts, as these actions can have direct implications under animal welfare and criminal laws [17].

The One Health concept [18], which encompasses the interconnectedness of human, animal, and environmental health, also extends to the public health implications of zoophilia or sexual contact with animals. Engaging in such activities presents several health risks, which depend on the level and form of contact involved. The literature typically categorizes these acts into five types: (1) genital acts, (2) oral genital acts, (3) masturbation, (4) frotteurism (rubbing genitals against animals), and (5) voyeurism (the observation of third parties during sexual intercourse with animals) [19]. The risks to both animal welfare and human health vary depending not only on the form of interaction but on the specific animal involved. Various paraphilias, such as canophilia (sexual attraction to dogs) and ailurophilia (sexual attraction to cats) [20], highlight the range of sexual attractions towards different animals. Animals can harbor numerous microorganisms that pose risks to humans, with zoonotic diseases, although infrequent, potentially transmitted through sexual contact (e.g., hookworm infections, chlamydia, salmonella) [21].

Internationally, legal responses to zoophilia vary significantly. In their 2005 review, Bolliger and Goetschel further established that the legal protection for sexually exploited animals was inadequate, revealing that such practices were more common than assumed and largely unpunished in many countries. They noted that, while perceptions of zoophilia as a legal issue had decreased since the Enlightenment, actions causing significant suffering to animals were still subject to penalties under animal welfare laws [22]. However, the past two decades have seen significant changes in this area, with some countries recriminalizing zoophilic acts. In countries like the Netherlands, Norway, and Switzerland, detailed criminal legislation specifically targets zoophilic acts, including the possession and distribution of animal pornography. For instance, Dutch law criminalizes sexual acts with animals and imposes penalties for related visual materials depicting such acts. Similarly, Swiss legislation incorporates a concept of animal dignity, prohibiting sexually motivated acts with animals irrespective of harm [11]. It is noteworthy to mention the legal changes concerning zoophilic acts in the United States. In a review conducted in 2014, the authors found that only 31 states had laws criminalizing bestiality, with 16 states imposing felonies and 15 imposing misdemeanors for such acts [23]. Since then, the legal landscape has changed dramatically, as all states except West Virginia now have statutes that impose sanctions for sexual acts with animals in the United States [24]. Contrastingly, in Italy, Slovenia, and Hungary, zoophilia lacks specific criminal sanctions, although general animal cruelty laws may address some aspects of this issue [11]. In Hungary, while the Animal Protection Act prohibits certain forms of animal cruelty and exploitation, including acts of zoophilia, it does not explicitly criminalize zoophilia within the Penal Code [25]. The current Hungarian legal framework includes administrative penalties related to animal protection [26] but falls short of imposing comprehensive criminal sanctions for zoophilic acts.

It is important to note that there is a growing trend of describing zoophilic acts as “animal sexual assault”, with a significant impact on the pressure to strengthen laws against such acts. According to a 1997 study, zoophilic acts should be understood as interspecies sexual assault, as the circumstances surrounding animals as abused victims resemble those of women and children. The author argues that human–animal sexual relations typically involve coercion, often inflict pain or death on animals, and lack any meaningful form of consent from the animals involved [27]. Similarly, a 2017 study emphasizes the lack of consent, concluding that all sexual advances toward animals should be classified as sexual assault since animals are unable to communicate consent in a way that humans can understand. The work discusses the prevalence of animal sexual assault, suggesting that studies indicate that up to 35% of adult populations have engaged in such acts, while acknowledging the methodological limitations of these findings [28]. Some authors specifically address crush videos, which are graphic recordings of animals being crushed or otherwise harmed for the purpose of sexual gratification. This subject falls within the broader context of animal sexual assault [29].

It is also crucial to note that zoophilic acts often serve as a predictor for other criminal offenses, and perpetrators of such acts are frequently observed to commit additional crimes, as well. Edwards (2019) conducted a quantitative, descriptive study examining 456 arrests for bestiality incidents in the U.S. from 1975 to 2015, revealing that animal sex offending was often correlated with other criminal behaviors. The findings indicate that 31.6% of animal sex offenders also abused children and adults, while 52.9% had prior criminal records related to sexual abuse or violence. Notably, only 39.1% of arrests for direct sexual abuse of animals led to prosecution, suggesting that bestiality was more pervasive than previously recognized, highlighting the need for further research to enhance legal responses [30].

Understanding public opinion regarding zoophilia is crucial in informing legal, ethical, and social discussions surrounding this sensitive issue. Accordingly, some studies have been conducted using mostly online methods to survey public attitudes toward this topic, as well as to explore the perspectives of zoophiles themselves. A 2003 study examined 114 self-identified zoophile men through an online questionnaire. The research explores how they acquired their zoophile identity and eroticized animals and presents their sexual profiles, highlighting the different forms of zoophilia [31]. In a 2018 study, researchers analyzed online communities of zoophiles, gathering responses from 138 participants. Key topics included the perception of animals as objects of love, beliefs about animals’ ability to consent to sexual behavior, and misconceptions regarding zoophilia and bestiality. Many participants also reported a lack of emotional support from family and faced widespread public contempt [32]. A 2019 study examined the community of zoophiles recruited from the internet. The main finding was that these individuals were aware of their sexuality and recognized that their behavior was illegal, yet they viewed it as an expression of love. They considered sexual experiences with animals to be of higher quality than those with humans, and a fundamental motivation for them was the desire for a long-term relationship with an animal partner [33].

There are a few studies that locally compare the legal regulation of zoophilia with public perception, such as a 2022 study focusing on Maltese legislation. This research aimed to analyze the conformity between public opinion and the current Maltese laws. Using a quantitative research approach, the study gathered data through an online survey. The findings reveal a disparity between public opinion and Maltese legislation, indicating that this subject has not been adequately addressed in Malta, necessitating further information and enlightenment for participants and stakeholders [34].

The aim of this study was to provide preliminary insights into Hungarian public perceptions and attitudes towards zoophilia and zoophilic acts. The study aimed to provide an understanding of the level of awareness regarding zoophilia, explore public attitudes toward its acceptability from health and animal welfare perspectives, and identify perceived contributing factors. Furthermore, it aimed to assess the level of public support for legal measures against zoophilia and to explore how demographic factors may influence opinions and knowledge on the subject. While the chosen methodology may not yield nationally representative results, it provides a valuable framework for investigating specific predictions. The study hypothesized that perceptions of zoophilia among respondents would be predominantly negative, reflecting societal and animal welfare concerns. It was also suggested that, based on the respondents’ views, criminalizing zoophilia could lead to improved animal protection. Individuals who keep pets were anticipated to express a greater degree of condemnation toward zoophilia, indicative of their increased awareness and sensitivity to the issue. Additionally, demographic factors such as gender, education, and residential environment were assumed to influence attitudes, with higher-educated urban women being overrepresented and more sensitive to the topic. Through this research, the study aimed to provide insights that could inform legal and educational interventions concerning animal protection and zoophilia.

## 2. Materials and Methods

This study was conducted in accordance with the Research Ethics Codex of the Hungarian Academy of Sciences; the survey was approved by the Scientific and Innovation Committee of the University of Veterinary Medicine, Budapest.

Specialized surveys were conducted through social media (Facebook, Menlo Park, CA, USA) [35] from 31 October to 31 December 2021. Participants took part in the survey voluntarily and remained anonymous, and were all above the Hungarian age of majority (18 years). Before the survey began, they provided their written consent to participate in the research. The anonymous online questionnaire was shared by the Centre for Animal Welfare of the University of Veterinary Medicine; therefore, it can be presumed that it reached a relatively high proportion of individuals interested in animal welfare. The invitation began with “a questionnaire about zoophilia that can be completed in 5–10 min”. Closed (Q1–Q5, Q7–Q8, Q11–Q13, Q15, Q18, Q20–Q22) and open-ended (Q6, Q9–Q10, Q14, Q16–Q17, Q19) questions were also used (Table 1).

The first group of questions (Q1–Q6) concerned demographic information (gender, age, education, the size of the place of residence, does the respondent keep animals, and if so, what kind of animals). The second group (Q7–Q22) concerned questions about knowledge (Q8, Q9, Q10), personal experiences (Q15–Q19), and opinions (Q11, Q12, Q14, Q20–Q22) about zoophilia and zoophilic acts and opinions about related concepts such as the dignity of animals (Q7, Q13).

### Statistical Analysis

The statistical analysis of the survey data was conducted using Microsoft Excel (Redmond, WA, USA) [36]. The independent variables included demographic factors such as gender, age, education level, place of residence, and animal-keeping status. Additionally, the analysis incorporated responses related to participants’ knowledge, experiences, and opinions regarding zoophilia and animal dignity. Conversely, the dependent variables encompassed beliefs about animal dignity, the acceptance of zoophilia concerning human health and animal welfare, and perceptions of the factors contributing to the emergence of zoophilia.

To summarize the data, descriptive statistics were computed, including the mean and median for continuous variables, along with percentages for categorical variables derived from survey responses. Several chi-square tests were conducted to assess the associations between independent variables and dependent beliefs, with a significance level set at *p* < 0.05. Results with *p* < 0.001 were considered highly significant. Furthermore, independent *t*-tests were performed to compare group means, with a significance threshold set at *p* < 0.05. For the open-ended responses regarding the factors contributing to zoophilia, qualitative analysis was performed to categorize responses into themes.

## 3. Results and Discussion

The questionnaire received responses from 174 male and 1579 female participants. This gender disparity may be attributed to several factors, including women tending to spend more time on social media than men. Their motivations for using these platforms often differ, with women having a stronger focus on social topics [37]. The gender disparity may also be explained by the fact that, due to the distribution platform and method, the online questionnaire likely reached a higher proportion of respondents interested in animal welfare. According to numerous studies, women are overrepresented among individuals with an interest in animal welfare; they are generally more responsive and engaged when it comes to participating in surveys related to animals [38,39]. For instance, a 1991 study involving 144 male and 222 female undergraduate students, who completed the Bem Sex-Role Inventory and a questionnaire on attitudes toward animal welfare and ethical issues, found that women exhibited significantly greater concern for animal welfare and ethical considerations compared to men [40].

The respondents were distributed across age groups as follows: 41.6% were aged 18–30 years, reflecting a strong representation of young adults; 24.6% belonged to the 31–40 age group, while 20.8% were middle-aged adults aged 41–50 years. Additionally, 9.2% were aged 51–60 years, and 3.9% were over 60 years old, with the oldest respondents being 77 years old. This age diversity underscores broad participation across different generations in the survey, with a mean age of 35.9 years and a median age of 34 years. In terms of education, 43.5% held higher education degrees, 44.7% had high school diplomas, and 9.1% had completed vocational training or specialized school, indicating an overrepresentation of these educational levels. In contrast, only 2.5% had completed only elementary school, with just 0.06% having less than eight grades of education. Regarding geographical distribution, the majority of the respondents—29.7%—came from the capital city, Budapest. This was followed by 23.5% from larger cities or county seats, 25.0% from small towns, and 20.6% from villages. Additionally, 1.1% were from farms or isolated rural properties.

Animal keepers were overrepresented among the respondents, likely due to their heightened sensitivity to issues related to animal welfare and zoophilia. Specifically, 1568 respondents indicated that they were animal keepers, compared to only 185 who were not. The most commonly kept animals are dogs and cats, with 1299 respondents indicating that they kept dogs and 692 kept cats. Birds were kept by 105 respondents; fish and other aquatic animals were also represented, with 74 respondents. There were 100 reptile and amphibian keepers, and 204 respondents kept small mammals like rabbits (106) or guinea pigs (40). Livestock was also represented, with, for example, chickens (48), guinea fowl (48), and pigs (21), totaling 181 livestock keepers.

**Figure 1:**
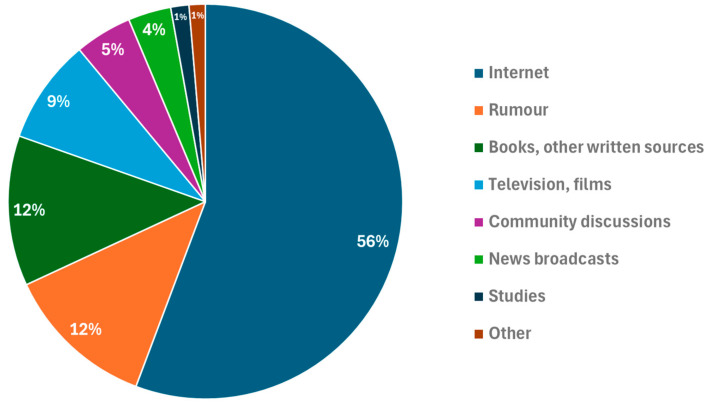
Sources of awareness about zoophilia among the surveyed Hungarian population (N = 1877).

To the question asking whether respondents had ever heard of zoophilia, a total of 84% of respondents answered “Yes,” while 16% answered “No”. When asked where they had heard about zoophilia (Q9, open question), the responses were varied. The majority of respondents, 56%, reported having learnt about it from the internet. Rumour was the second most common source, cited by 12% of the respondents, followed closely by reading books (including the Bible and Gabriel García Márquez’s book *One Hundred Years of Solitude*), newspapers, and other written sources, which 12% of the respondents mentioned. Other notable sources included television and films (9% in total), community discussions (5%), and news broadcasts (4% of the respondents). Additionally, 1% referred to different studies, including university courses, such as psychological, veterinary, and legal studies. The ’Others’ category, which includes less conventional sources (for example, jokes or a ’former spouse’s computer’), accounted for 1% of the respondents (Figure 1).

According to the survey, out of 1512 respondents, 1.7% believed that zoophilia is acceptable from the perspective of human health, while 98.3% disagreed. Among the 26 respondents who answered “yes”, 9 were male, representing 34.6% of this group. This is notable when compared to the overall sample, where males comprised 9.9% (174 out of 1753 respondents). According to the survey, out of 1515 respondents, only 1.7% believed that zoophilia was acceptable from the perspective of animal welfare, while 98.3% disagreed.

Among those who believed that zoophilia was acceptable from the perspective of animal welfare, 12 individuals (48%) also thought it was acceptable from the perspective of human health. Of these, ten respondents (40%) believed that it did not negatively impact the dignity of animals, either. Among these ten respondents, five cited psychological state as a factor in the development of zoophilia (Q14). One person referred to taste, another to trauma, one to necessity, one to upbringing, and one to age, although it is important to note that the low number of respondents in this segment may have led to lower relevance in the findings. Out of the ten respondents mentioned, two reported that zoophilic incidents occurred in their environment or that acquaintances had told them about such cases (Q15). One respondent mentioned only that the incident involved a fox and a dog and resulted in harm to the animals. The other provided a more detailed description, stating that a “22-year-old university student, normal but sometimes eccentric”, once engaged in sexual activity with a cow, which showed no external signs of harm. The respondent sharing this story agreed that zoophilic acts should be criminalized in Hungary (Q22).

**Figure 2:**
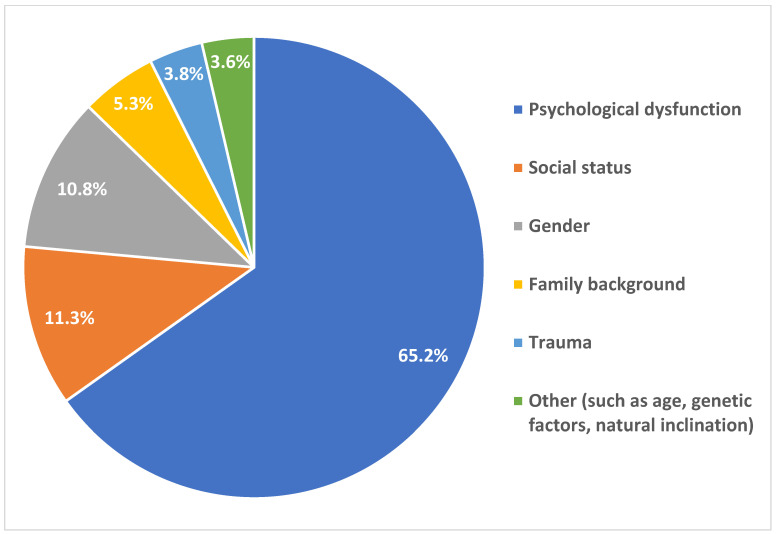
Factors contributing to the emergence of zoophilia according to the surveyed Hungarian population (N = 1438).

A total of 1438 responses were provided; when respondents were asked about the factors that might contribute to the development of zoophilia, such as age, gender, social status, and psychological dysfunction, they could also mention other factors (Q14). This was an open-ended question, and a qualitative analysis was performed to categorize the responses into themes. The “psychological dysfunction” category included all responses that, in any way, suggested that the cause of zoophilic acts is related to some form of psychological or psychiatric disorder or issue. The majority of respondents, 1126 people (65.2%), believed that some form of psychological dysfunction plays a significant role in the development of zoophilia. Social status was identified by 11.3% of respondents, and gender was mentioned by 10.8%. Trauma was considered a contributing factor by 3.8%. Smaller groups of respondents cited family background, including upbringing and being single, totaling 5.3%. Additionally, an “Other” category was created, which includes factors such as age, genetic factors, or natural inclination (3.7%) (Figure 2). These findings align with the broader understanding that mental health plays a crucial role in such behaviors, though other social factors are also relevant [41].

These results indicate that psychological state is perceived as the most significant factor in the development of zoophilia, with other factors such as social status, gender, and trauma also considered relevant, although to a lesser extent. The diversity in responses highlights a broad range of opinions on what influences the development of zoophilia.

According to the research findings, out of a total of 1753 respondents, 98.9% believe that animals have dignity (Q7), while 1.1% do not share this belief. This overwhelming majority underscores a strong societal consensus on animal dignity. Examining demographic factors, we can see that among the 20 non-believers, 35% are male, whereas males comprise 9.9% of the entire sample. Additionally, 60% of non-believers have a college degree or higher, compared to 43.5% of the total sample having such qualifications. Notably, there are also respondents with primary education (one individual) or less (also one individual) among non-believers, highlighting varied educational backgrounds within this group; however, the very low sample size limits the ability to draw more robust conclusions. Among those who believe that animals have dignity (those who answered “yes” to Q7), 84.7% think that zoophilia negatively affects the dignity of animals, while 2% do not believe that it has a negative impact.

A significant association was observed between animal keeping (Q5) and belief in animal dignity (Q7), as indicated by a chi-squared test (χ^2^ = 12.81, df = 1, *p* < 0.001), which was significantly below the 0.05 significance level. This suggests that individuals who engage in animal keeping are more likely to believe in animal dignity. This finding highlights the potential role of proximity to animals in shaping attitudes towards animal sentience. Research indicates that attitudes towards animals are strongly influenced by whether individuals keep animals and their exposure to them. Non-keepers attribute fewer emotions to animals compared to keepers [42].

**Figure 3:**
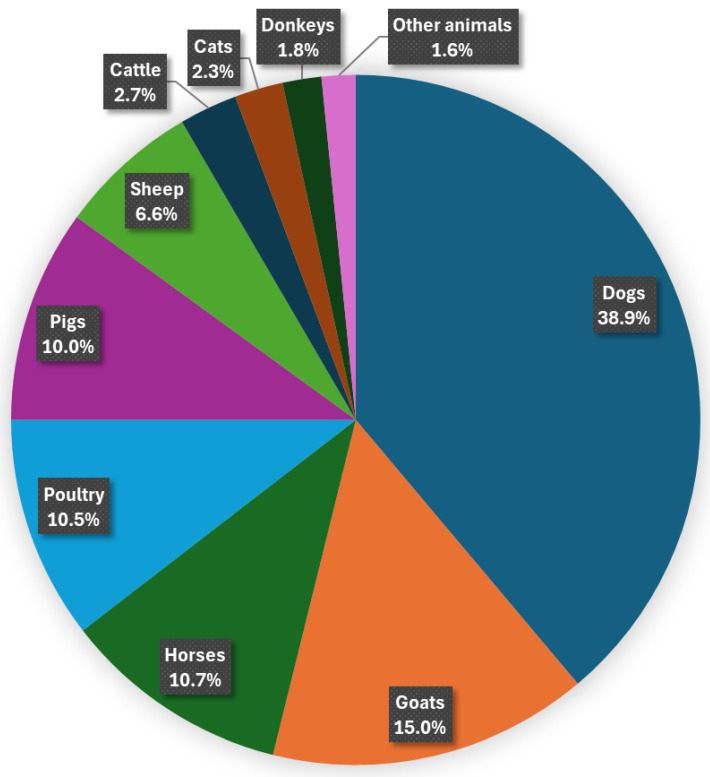
Distribution of animals involved in zoophilic incidents based on 440 cases, categorized by species.

Out of the total respondents to Q15, 14% reported that they had encountered a zoophilic case in their environment or heard about such an incident from acquaintances. In contrast, 86% stated that they had not come across any zoophilic cases. A total of 440 animals were mentioned in relation to zoophilic incidents (Q16). Of these, the biggest group, 38.9% were dogs, 15.0% were goats, and 10.7% were horses. Poultry, including chickens, ducks, geese, roosters, turkeys, and pigeons, were the most frequently mentioned, followed by pigs. Sheep were mentioned less frequently than both poultry and pigs. Cattle and cats appeared less often, with donkeys being the least frequently mentioned among the animals discussed. Other animals, including snakes, fish, and miscellaneous livestock, comprised 1.6% of cases (seven cases) (Figure 3). Lorászkó et al. (2021) investigated 591 final verdicts on animal cruelty in Hungary. Concerning the percentage distribution of species involved in court proceedings from 2004 to 2019, it was also found that most cases of animal cruelty involved dogs (73%). However, in terms of the species affected by animal cruelty, cats ranked second (10%), followed by other livestock (such as cattle and pigs) (9%) and wild animals (4%) [43].

**Figure 4:**
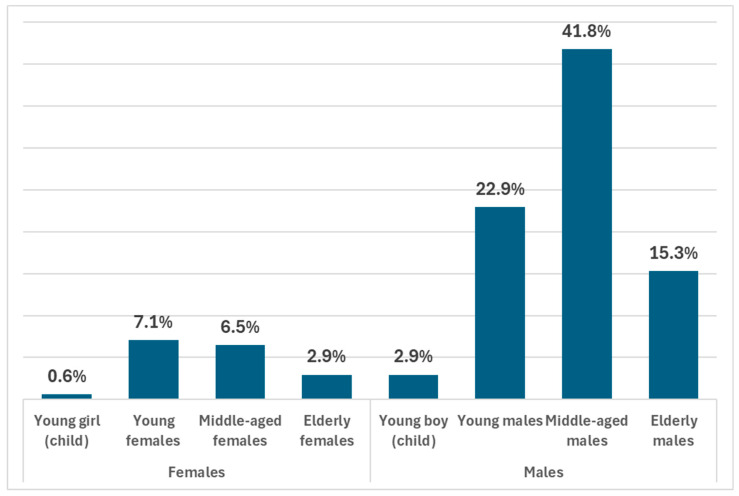
Distribution of individuals involved in zoophilic acts by age and gender based on 170 mentions from 363 survey responses.

In response to question Q17, which was an open-ended inquiry regarding the characteristics of individuals involved in zoophilia, 363 responses were given. These responses associated a total of 170 individuals with zoophilic acts. Males were mentioned about 4.9 times more frequently than females. Among males, middle-aged males were the most frequently referenced group, followed by young males and then elderly males. Regarding females, young females were mentioned most often, followed by middle-aged and elderly females (Figure 4).

This result is consistent with other research examining gender distribution in zoophilia. The Hunt study (1974), based on responses from 982 men and 1044 women to sex information questionnaires, reported that 4.9% of men and 1.9% of women admitted to engaging in bestiality [44]. According to Sendler (2019), who administered a survey via the internet on popular discussion forums targeting communities of zoophiles, the gender distribution showed that 72% of the sample were males and 28% were females. The largest age group was 20–25 years old. The next most common groups were those aged 25–30 and 30–40, with a mean age of 36.7 years [33].

Regarding substance use, 6% of the 363 responses mentioned drug use, and 18% cited alcohol use. In terms of education and intelligence, 46% of respondents characterized the individual as having low education, while 26% described the person as intelligent. When it came to mental health, 57% of the responses indicated that the person was psychologically ill. Finally, 7% mentioned childhood neglect or bad examples as factors.

**Figure 5:**
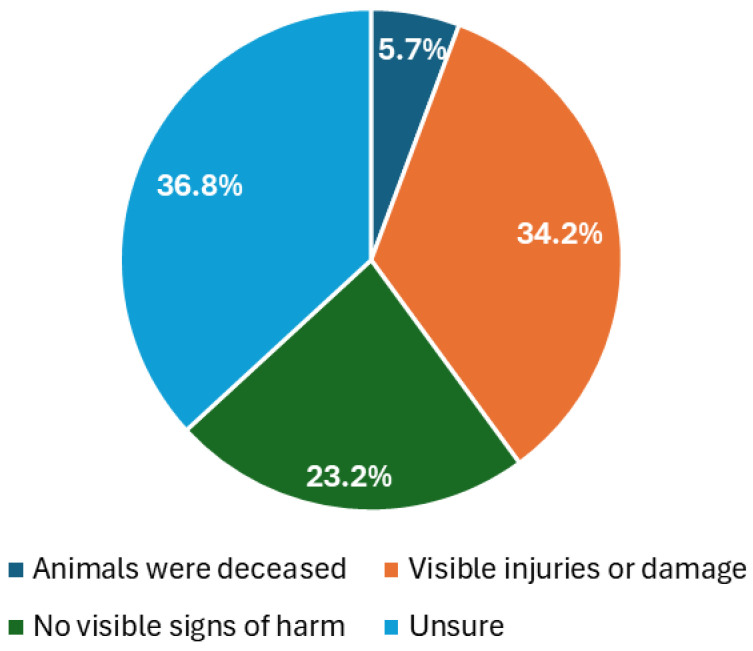
Condition of animals involved in zoophilic acts (N = 228).

Of the 228 responses concerning the condition of animals involved in zoophilia, 34.2% reported visible injuries or damage, 23.2% saw no signs of harm, 36.8% were unsure, and 5.7% noted that the animals were deceased (Figure 5).

The open-ended question Q19, which asked about the circumstances of zoophilic incidents that the respondents had heard of, received a total of 184 responses. Five comments described how such incidents were uncovered by neighbors. There were also calls for stronger animal protection laws, psychological interventions, and increased awareness. Three respondents expressed skepticism about the accuracy of reported incidents.

**Figure 6:**
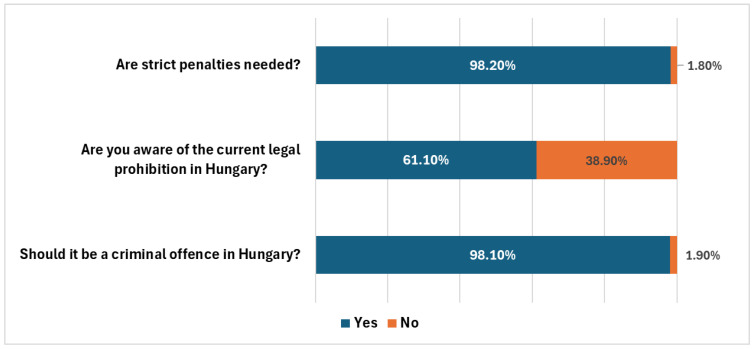
Hungarian respondents’ attitude toward, and awareness of, animal cruelty laws and penalties (answers to Q20, Q21, and Q22; N = 1753).

In response to whether those who use animals for sexual gratification should face strict penalties (Q20), the great majority (98.2% of respondents) agreed, and 61.1% of respondents were aware of the current Hungarian law prohibiting the use of animals for acts aimed at sexual gratification, while 38.9% were not (Q21), which means that many people were unaware of the legal prohibition of zoophilic acts in Hungary. Regarding whether it should be a criminal offense in Hungary to use animals for sexual gratification (Q22), again, the vast majority (98.1% of respondents) believed that it should be (Figure 6). These results underscore the need for enhanced public education on legal standards regarding zoophilic acts and reflect a robust societal consensus favoring strict penalties for zoophilic acts.

Based on the research findings, among demographic factors, gender and settlement type had a greater influence on attitudes toward zoophilic acts than age and educational level. No significant difference in age (Q2) was found between those who agree and those who disagree with strict sanctions for using animals for sexual purposes (Q20) (*t*-test result: *t* = 0.0902, df = 1751, *p* = 0.928). Additionally, no significant association was found between educational level (Q3) and the preference for strict sanctions (Q20) (chi-squared test result: χ^2^ = 1.48, df = 4, *p* = 0.829). Neither age (Q2) nor educational level (Q3) was significantly associated with whether respondents knew that current Hungarian law prohibited using animals for sexual gratification (Q21) (*t*-test result for age: *t* = 1.554, df = 1751, *p* = 0.120, chi-squared test result for educational level: χ^2^ = 8.52, df = 4, *p* = 0.074).

However, attitudes toward zoophilic acts showed significant gender differences. Women were significantly more aware of the legal prohibition (Q21) than men (chi-squared test result: χ^2^= 5.12, df = 1, *p* = 0.024) and were significantly more likely to support strict sanctions for zoophilic acts (Q20) than men (chi-squared test result: χ^2^ = 29.24, df = 1, *p* < 0.001). Additionally, women were significantly more likely to consider zoophilic acts a crime (Q22) compared to men (chi-squared test result: χ^2^ = 31.1, df = 1, *p* < 0.001).

A highly significant association (chi-squared test result: χ^2^ = 20.84, df = 4, *p* < 0.001) was found between settlement type (Q4) and awareness of the legal prohibition on using animals for sexual gratification (Q21). People in capital cities and major cities were generally more aware of the law compared to those in smaller towns, villages, and rural areas. Additionally, a significant association (chi-squared test result: χ^2^ = 15.42, df = 4, *p* = 0.004) was found between settlement type (Q4) and opinions on imposing strict sanctions for zoophilic acts (Q20); residents of capital cities and major cities generally showed strong support, while those in smaller towns and villages also favored strict sanctions but exhibited varying levels of opposition.

The research provided support for the predictions outlined in the predictions. The first assumption, which posits a predominantly negative perception of zoophilia, was clearly validated, as a striking 98.3% of respondents deemed such behavior to be unacceptable, demonstrating a societal consensus that condemns zoophilia from both human health and animal welfare perspectives. The second prediction, suggesting that criminalizing zoophilia could enhance animal protection, was also substantiated, given that a significant majority of participants expressed support for penalties against individuals involved in such acts, reflecting a societal demand for legal measures to safeguard animal welfare. Furthermore, the third assumption, which indicated that pet keepers condemn zoophilia more strongly, found support in the data, as individuals who kept pets—a substantial portion of respondents—demonstrated heightened sensitivity to animal welfare issues and a clear rejection of zoophilic acts. Finally, the prediction regarding the influence of demographic factors on attitudes toward zoophilia was confirmed. The respondent pool was predominantly female, and the correlation between higher education levels and belief in animal dignity, coupled with an overrepresentation of educated urban respondents, suggests that these demographic characteristics play an important role in shaping public opinion on zoophilia. Ultimately, the findings collectively affirm a societal readiness to address zoophilia as a serious issue, while reflecting an evolving understanding of animal welfare in Hungary.

## 4. Conclusions

This research confirms that zoophilia remains an underexplored and taboo subject, despite its implications for human and animal health, as well as animal welfare. The persistent stigma surrounding zoophilia has led to insufficient attention from both scientific and legislative perspectives, resulting in gaps in our understanding and management of the associated issues in Hungary and some other countries.

The study revealed a strong public consensus supporting the recognition of animal dignity (at least in a moral sense) and the implementation of stricter penalties for zoophilic acts. Despite this, a significant number of respondents were unaware of the existing legal framework in Hungary, which points to an urgent need for improved public education and outreach. Future campaigns should focus on increasing public knowledge on animal welfare laws and the ethical concerns surrounding zoophilia.

Additionally, the findings indicate that gender and settlement type significantly influence attitudes toward zoophilia. These results suggest that future research should focus on understanding the underlying factors contributing to these demographic differences. It is important to develop tailored educational campaigns and targeted policy interventions to address these variations effectively. Enhancing public awareness and engagement through such focused efforts could help bridge the gaps in understanding and support across different communities.

The study overall underscored the need for more research into zoophilia and particularly into how different legal systems handle it and where reforms are needed. It is also crucial to investigate health risks, such as zoonotic diseases and psychological effects, to develop preventive and treatment strategies. Furthermore, understanding the psychological and social impacts on the animals and individuals involved could improve both legal and health responses. This research will help inform policymakers and health professionals, leading to more effective frameworks and strategies.

## Figures and Tables

**Table 1:** Questions from the survey on the Hungarian public’s perception of zoophilia and zoophilic acts.

Q1	What is your gender?
Q2	What is your age?
Q3	What is your highest level of education?
Q4	What is the type of your place of residence?
Q5	Do you keep animals?
Q6	If you keep Animals (i.e., You answered “Yes” to Q5), what type(s) of animals do you keep at home?
Q7	Do you think animals have dignity?
Q8	Have you heard about zoophilia before? (Zoophilia is the sexual attraction of humans towards animals or sexual activities involving animals)
Q9	If you have heard about Zoophilia (i.e., You answered “Yes” to Q8), where have you heard or learned about zoophilia? (e.g., Internet, community, word of mouth, readings, etc.)
Q10	If you have heard about Zoophilia (i.e., You answered “Yes” to Q8), what have you heard about it? (Please summarize in one or two sentences)
Q11	Do you think zoophilia is acceptable from the perspective of human health?
Q12	Do you think zoophilia is acceptable from the perspective of animal welfare?
Q13	If you believe that animals have Dignity (i.e., You answered “Yes” to Q7), do you think zoophilia negatively affects the dignity of animals?
Q14	What factors do you think might contribute to the emergence of Zoophilia (e.g., Age, gender, social status, psychological condition)
Q15	Have you ever encountered a case of zoophilia in your environment, or have your acquaintances told you about such an incident?
Q16	If you have already encountered a case of zoophilia in your Environment (i.e., You answered “Yes” to Q15), what type of animal was involved in the zoophilic Incident (e.g., Species, size, age, other characteristics)?
Q17	If you have already encountered a case of zoophilia in your Environment (i.e., You answered “Yes” to Q15), how would you describe the person involved in the zoophilic Act (e.g., Gender, age, education, psychological condition)?
Q18	If you have already encountered a case of zoophilia in your Environment (i.e., You answered “Yes” to Q15), to your knowledge, did the animal involved in the zoophilic act show any signs of physical harm or suffer any other damage?
Q19	If you have already encountered a case of zoophilia in your Environment (i.e., You answered “Yes” to Q15), are there any other circumstances related to the incident that you consider important to mention?
Q20	Do you agree that anyone using an animal for sexual gratification should face penalties?
Q21	Did you know that, according to current Hungarian laws, it is prohibited to use an animal for acts aimed at sexual gratification?
Q22	Do you think it should be a criminal offense in Hungary to use an animal for sexual gratification (as currently, the Penal Code does not include such an offense)?

## Data Availability

The data presented in this study are available on request from the corresponding author without undue reservation.
